# Supplementary material for: The Upregulation of Leucine-Rich Repeat Containing 1 Expression Activates Hepatic Stellate Cells and Promotes Liver Fibrosis by Stabilizing Phosphorylated Smad2/3
Source: Int J Mol Sci. 2024 Feb 27;25(5):2735. doi: 10.3390/ijms25052735 (PMC10932271; doi:10.3390/ijms25052735)
Supplement: Supplementary file 1 [file ijms-25-02735-s001.zip › ijms-2868253-supplementary.pdf]

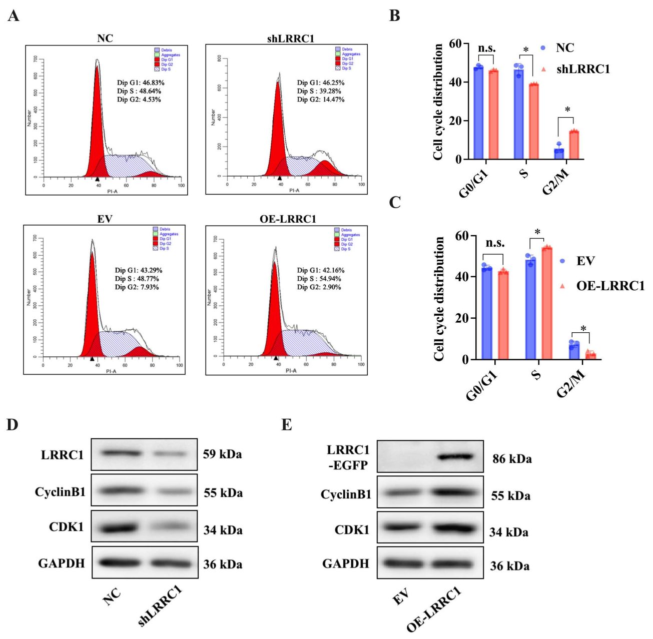

Supplementary Figure 1 Effects of LRRC1 on the cell cycle phase distribution of LX-2 cells. (A) Fluorescence-activated cell sorting (FACS) analysis of the cell cycle distribution of LRRC1-knockdown or -overexpressing LX-2 cells. (B-C) Comparison of the cell cycle distribution of LRRC1-knockdown (B) or LRRC1-overexpressing (C) LX-2 cells. (D-E) Western blot analysis of the expression of cell cycle regulatory proteins Cyclin B1 and CDK1 in LRRC1-knockdown (D) or -overexpressing (E) LX-2 cells. The data in B and C were analyzed by Student's t test and are presented as means ± SDs. n.s., not significant; **P* < 0.05, ***P* < 0.01, ****P* < 0.001.

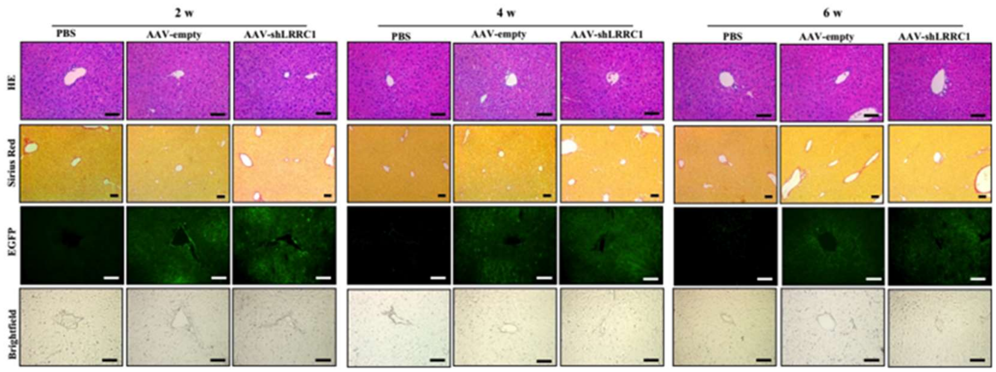

Supplementary Figure 2 Efficiency of pAAV-U6-shLRRC1-CMV-EGFP injection. Representative images of HE staining (scale bars, 200 μm), Sirius Red staining (scale bars, 100 μm) and EGFP fluorescence (scale bars, 200 μm) in liver tissues from the AAV-empty group, AAV-shLRRC1 group, and blank control group (PBS).

Supplementary Table 1 Primer sequences used for plasmid construction

Forward primer (5′→3′)	Reverse primer (5′→3′)
CTAGAGGAACTGAGAGAGAATCTTCTCTTTCCTTTTCGAGAGAAGATTCTCTCTCAGGA	CTAGTCAAAAAGGAACTGAGAGAGAATCTTCTCTCGAGAGAAGATTCTCTCTCAGTTCCT-3

Supplementary Table 2 Primer sequences used for qRT-PCR

Species	Gene	Forward primer (5′→3′)	Reverse primer (5′→3′)
Mouse			
	α-SMA	TCAGGGAGTAATGGTTGGAATG	GGTGATGATGCCGTGTTCTA
	LRRC1	AGATCTACCGCTATGCCCGC	GCTATTTCTGGAGGGAGCCG
	Col-I	AGACCTGTGTGTTCCCTACT	GAATCCATCGGTCATGCTCTC
	Col-III	CTGTAATATGGAAACCGGAGAAA	CCATAGCTGAACTGAAAGCCACC
	GAPDH	GCACAGTCAAGGCCGAGAAT	GCCTTCTCCATGGTGGTGAA
Human			
	LRRC1	TCCTTACCAAAAGAGATCGG	GGTAGATGCAGCAACCTGT
	GAPDH	GCACAGTCAAGGCCGAGAAT	GCCTTCTCCATGGTGGTGAA
